# The phylogeny of the mammalian heme peroxidases and the evolution of their diverse functions

**DOI:** 10.1186/1471-2148-8-101

**Published:** 2008-03-27

**Authors:** Noeleen B Loughran, Brendan O'Connor, Ciarán Ó'Fágáin, Mary J O'Connell

**Affiliations:** 1 Bioinformatics and Molecular Evolution Group, School of Biotechnology, Dublin City University, Glasnevin, Dublin 9, Ireland; 2 School of Biotechnology, Dublin City University, Glasnevin, Dublin 9, Ireland

## Abstract

**Background:**

The mammalian heme peroxidases (MHPs) are a medically important group of enzymes. Included in this group are myeloperoxidase, eosinophil peroxidase, lactoperoxidase, and thyroid peroxidase. These enzymes are associated with such diverse diseases as asthma, Alzheimer's disease and inflammatory vascular disease. Despite much effort to elucidate a clearer understanding of the function of the 4 major groups of this multigene family, we still do not have a clear understanding of their relationships to each other.

**Results:**

Sufficient signal exists for the resolution of the evolutionary relationships of this family of enzymes. We demonstrate, using a root mean squared deviation statistic, how the removal of the fastest evolving sites aids in the minimisation of the effect of long branch attraction and the generation of a highly supported phylogeny. Based on this phylogeny we have pinpointed the amino acid positions that have most likely contributed to the diverse functions of these enzymes. Many of these residues are in close proximity to sites implicated in protein misfolding, loss of function or disease.

**Conclusion:**

Our analysis of all available genomic sequence data for the MHPs from all available completed mammalian genomes, involved sophisticated methods of phylogeny reconstruction and data treatment. Our study has (i) fully resolved the phylogeny of the MHPs and the subsequent pattern of gene duplication, and (ii), we have detected amino acids under positive selection that have most likely contributed to the observed functional shifts in each type of MHP.

## Background

Heme peroxidases are readily abundant enzymes that can be classified into two major families, namely the animal and non-animal peroxidases, that have arisen from two independent evolutionary events [1]. The non-animal peroxidases include plant, bacterial, fungal and protist [1]. The classical peroxidase cycle involves the reaction sequence from native enzyme through compound I, then compound II and finally back to native enzyme [2]. An alternative and highly important pathway that mammalian heme peroxidases (MHPs) pass through, depending on substrate availability, is the halogenation cycle [3]. In the presence of H_2_O_2 _and a halide (especially iodide), myeloperoxidase (MPO) can catalyse a halogenation reaction that plays an important role in the antibacterial activity of leukocytes [4]. Animal peroxidases are a medically important group of enzymes implicated in many different diseases including asthma [5], Alzheimer's disease (AD) [6] and inflammatory vascular disease [7]. From biochemical studies it is believed that the heme peroxidases for mammals arose following a number of gene duplication events [3,8,9].

Gene duplication provides the raw material for evolution of diversity and is believed to be the principal source of new genes [10]. The process of gene duplication has a number of alternative outcomes, and remains a controversial issue. Gene duplicates may become functionally redundant [11], or functionally divergent. There are a number of ways in which functional redundant duplicates can be preserved [12,13]. It has been proposed that the preservation of duplicates can be brought about by degenerative mutations in the regulatory elements of the duplicates, this is referred to as the Duplication-Degeneration-Complementation model (DDC) [13]. The DDC model does not allow a role for positive selection in the evolution of duplicates and is based solely on a neutral model with degenerate mutations and subsequent negative selection. Under this model duplicates are preserved as each accumulates degenerate mutations, resulting in specific subfunctions that *in toto *ensure optimal fitness [13].

An alternative mode of duplicate retention is positive selection. For example, in direct contrast to the predictions of the DDC model it has been shown for human and mouse that the number of retentions and losses of duplicates fits more consistently with a model incorporating positive selection [14]. Rapid divergence in gene expression profiles of duplicates following the duplication event results in expression profiles as diverse as those of singletons. An example of this is the functional redundancy of transcription factor inhibitors, Iκα and β, that have acquired different functions through divergence of gene expression rather than biochemical function [15]. Recent studies have indicated that for mammalian genomes neofunctionalisation, be it independent of -, or coupled with – subfunctionalisation, is the most common mode of evolution of gene duplicates [16]. These selective pressures following the process of gene duplication are key to the evolution of specificity of divergent multigene families, such as the MHPs [17].

In those cases where having all duplicates is deleterious, dosage requirements may cause the partitioning of subfunctions to be favored by positive selection resulting from selective pressure for the fixation of nonfunctional or subfunctional alleles. The divergence of function may occur through neofunctionalisation [18], or, subfunctionalisation where the ancestral function is partitioned between the duplicates [19] (for detail on current gene duplication models see [20]).

We hypothesise that the selective pressures on MHPs following gene duplication events will, (i) still be traceable in the extant sequences of these enzymes, and (ii), will have contributed to the functional diversity observed in these enzymes. A fully resolved phylogeny can provide a basis for such comparative genomic analysis of these heme peroxidases.

MHPs have been classified into four main families based on their function; myeloperoxidase (MPO), eosinophil peroxidase (EPO), lactoperoxidase (LPO) and thyroid peroxidase (TPO). MPO, EPO and LPO function in antimicrobial and innate immune responses [21-23], whereas, TPO plays a key role in thyroid hormone biosynthesis [24], see Table 1. A study of the structure-function relationships of human heme peroxidases suggest that the evolution of TPO succeeded that of MPO, EPO and LPO, but that these families shared a common ancestor [3,8,9]. MHPs are present in various tissues and as such their peroxidase function varies depending on tissue of expression. There are both structural and functional similarities among this multigene family of enzymes particularly with respect to their catalytic domains, this reflects their evolutionary relatedness. It has been shown that active site residues are conserved in all heme peroxidases [3,25].

**Table 1:** Mammalian heme peroxidase features and functions (adapted from Clark 2000 and O'Brien 2000).

Superfamily (EC no.)	Chromosomal Location (Human)	Tissue Expression	Biological Function
MPO (1.11.1.7)	17	Neutrophils, mono-nuclear phagocytes	Microbicidal activity
EPO (1.11.1.7)	17	Eosinophils	Microbicidal activity
LPO (1.11.1.7)	17	Milk, saliva, tears and other secretions	Bacteriostatic and bactericidal activity
TPO (1.11.1.8)	2	Thyroid cell surface and cytoplasm	Thyroid hormone biosynthesis

MPO = Myeloperoxidase; EPO = Eosinophil peroxidase; LPO = Lactoperoxidase; TPO = Thyroid peroxidase.

To infer the phylogeny of the MHPs from sequence data, it is fundamental to consider the challenges associated with resolving mammalian gene phylogenies. The main pitfalls include poor phylogenetic signal resulting from mutationally saturated positions, inadequate modelling of the evolutionary process and systematic bias due to variable rates of evolution among species or within sequences [26].

A systematic bias or systematic error is one that results in greater support for an incorrect conclusion with the accumulation of more data. Long branch attraction (LBA) is one of the most commonly occurring systematic biases and is a consequence of unequal evolutionary rates across lineages. This can occur due to the number of cell divisions per unit time being different in different species or due to rapid fixation of mutations due to reduced population size, e.g., a bottleneck. Rodent species accumulate many more mutations within a defined time frame than larger mammals [27,28]. Therefore, rodentia are often placed close to the outgroup species on a phylogeny due to their increased number of mutations. There are a number of ways in which the noise (LBA) can be minimised. Firstly, the addition of more taxa to the dataset: denser sampling of species of intermediate generation time can reduce the effect of LBA by reducing the overall distances between taxa. Secondly, the use of improved models of sequence evolution, i.e., models sensitive to multiple substitutions at the same site and rate heterogeneity across the phylogeny. And finally, stripping the alignment of its most rapidly evolving sites and using only the remaining more slowly evolving sites to reconstruct phylogenies reduces the amount of LBA noise in the dataset [29]. These approaches can be used in combination. While databases such as Peroxibase [30] house all the up-to-date peroxidase sequences [31], we have included only those MHPs from completed mammalian genomes (allows us identify species-specific gene birth and death). We have used Maximum Likelihood (ML) and Bayesian methods of phylogeny reconstruction together with the stripping of the most rapidly evolving sites in the dataset.

The major questions addressed in this study pertain firstly to the resolution of the evolutionary relationships of these MHPs using molecular sequence data, and secondly, to the analysis of functional diversities among these superfamilies using the resolved phylogeny and ML methods for testing selective pressures.

Selection can be classified as being neutral, purifying or positive. Positive selection/Adaptive evolution is strongly indicative of functional shifts within proteins [32]. To determine what selective pressures may have influenced the functional diversification of the MHP families, we tested the data using a variety of ML models of evolution with different properties. These included models that allow for only purifying selection and/or neutral evolution, and those that allow for positive selection. Likelihood scores for all alternative models and their null hypotheses are calculated. The likelihood scores for the null hypothesis versus the alternative hypothesis for those models that are extensions of each other were then compared using a likelihood ratio test (LRT) for goodness-of-fit. For those models that allow for the estimation of site-specific evolution, we can identify those amino acids that have undergone positive selection. The location of these amino acid positions were estimated using Bayesian statistics and their location and possible functional significance were determined. In our analysis we have shown that positive selection has contributed to the evolution of these enzymes following gene duplication events.

## Results

### Phylogeny Reconstruction

**Figure 1:**
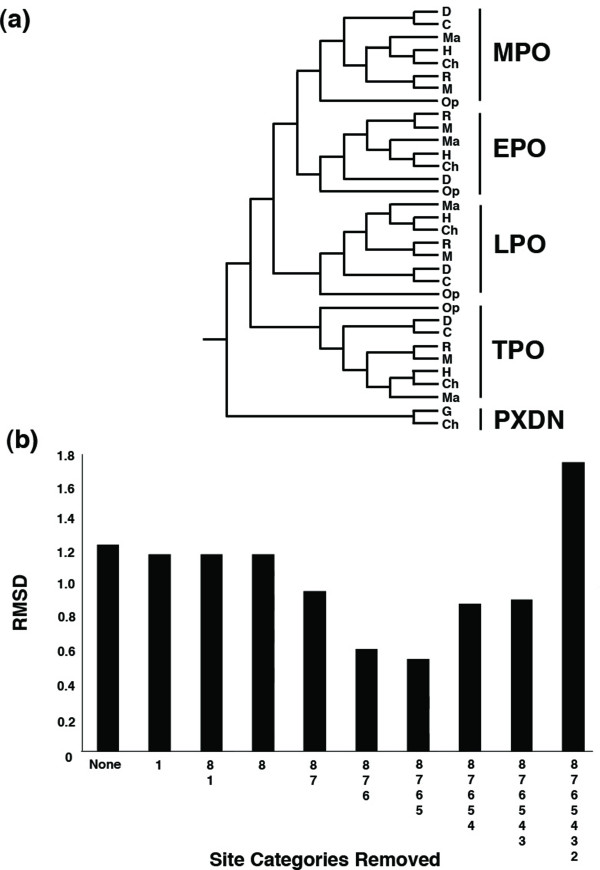
**The distance between each of the site stripped phylogenies and the ideal mammalian peroxidase phylogeny**. **(a) **The ideal phylogeny pruned from the mammalian phylogeny by Murphy *et al. *(2001), the peroxidasin sequences are outgroups to the MHP clade. The following are the species abbreviations used: Dog (D); Cow (C); Macaque (Ma); Human (H); Chimp (Ch); Rat (R); Mouse (M), Chicken (G), and Opossum (Op). This phylogeny was compared to each of the resultant site stripped phylogenies. **(b) **Graph showing the RMSD nodal distance (*y-axis*) between each site-stripped phylogeny (*x-axis*) and the ideal phylogeny. *On the X axis*: All: refers to the complete MSA; 8: site category 8 removed from the MSA; 8, 7: categories 8 and 7 removed from the MSA and so on up to the final column that contains only the most slowly evolving category of site. Values close to/zero correspond to complete agreement between the ideal and site stripped phylogeny.

**Figure 2:**
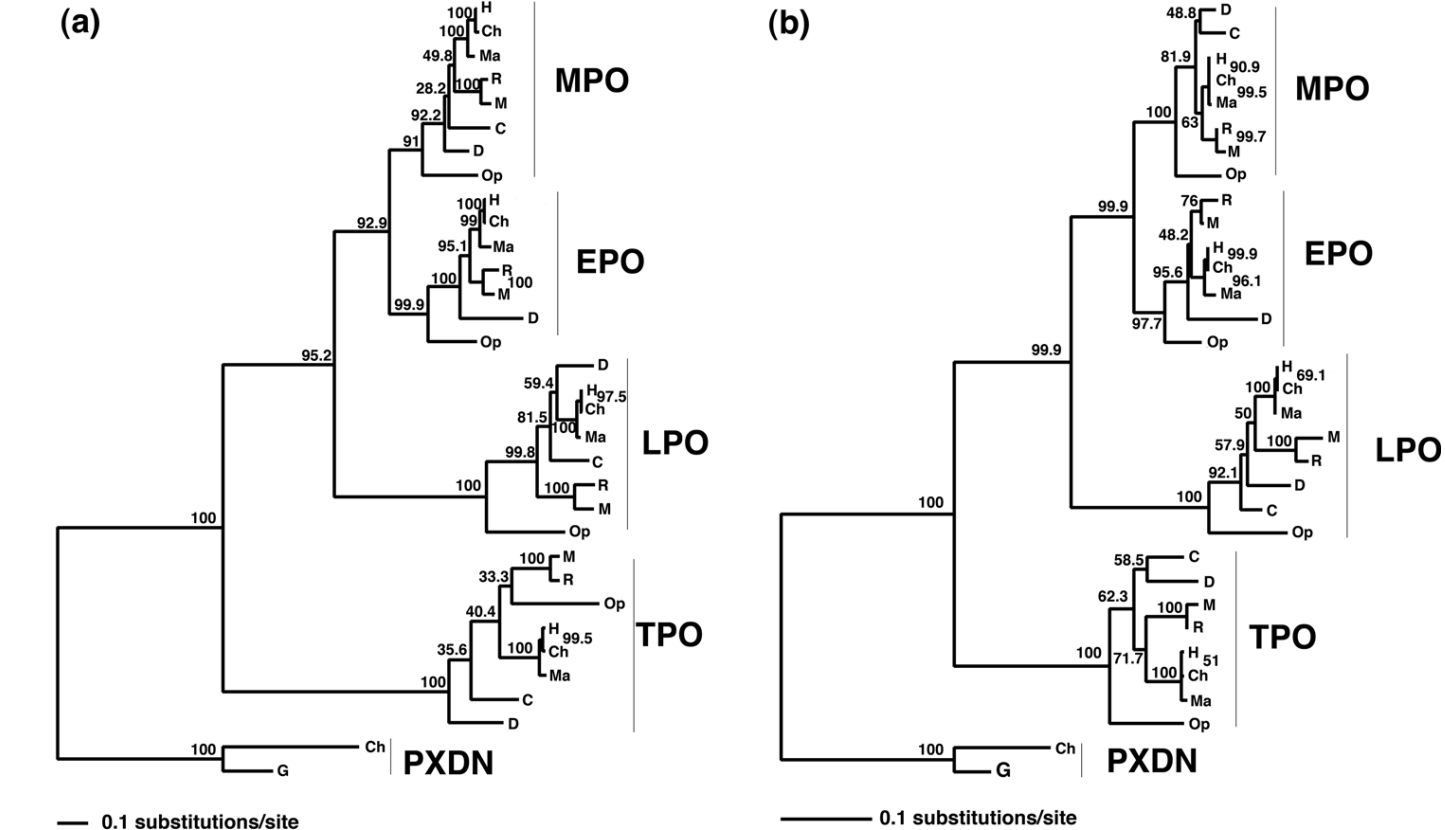
**Phylogeny of the mammalian heme peroxidases before treatment for long branch attraction and after treatment**. (a) Initial unresolved ML tree for mammalian heme peroxidases and peroxidasin from *Pan troglodytes *and *Gallus gallus *from the entire dataset. The bootstrap support values from 1000 replicates are shown on all nodes. (b) Resolved phylogeny following site stripping, the cow sequence for LPO can be seen to take an unusual place on the phylogeny.

The MHP dataset for this study consisted of 31 single gene orthologues from MPO, EPO, LPO, and TPO classes, totaling 1,017 aligned positions. The species phylogeny for the mammals has previously been fully resolved [33]. In brief, the mammalian species phylogeny describes Marsupialia (i.e. Opossum in our dataset) as outgroup to all other mammals, followed by the divergence of the Carnivora (i.e. Dog in our dataset) and the Cetacea (i.e. Cows in our dataset), and finally the emergence of the Euarchontoglires clade (i.e. primates and rodents) [33], see Figure 1a. The ML phylogenetic tree was estimated using MultiPhyl [34] and MrBayes 3.1.2 [35], the results were congruent, see Figure 2a. Each of the four superfamilies branched into their respective functional groups, with the members of the TPO superfamily taking the position of outgroup with high support values. The topology shows MPO, EPO and LPO shared a most recent common ancestor (MRCA) with a gene duplicate of TPO. The MPO and EPO groups themselves shared a MRCA and functionally diverged following a further gene duplication event. Therefore these two peroxidases (MPO & EPO) are the most closely related of all the MHPs in this study.

Despite the 4 major clades in the phylogeny corresponding to the 4 major groups of MHPs, the relationships of the species within these clades conflicts with the previously published mammalian species phylogeny [33]. The rat and mouse are members of the glires group, and as such are a sister group to the primates, which together form the Euarchontoglires mammalian superorder. The topology seen here for the LPOs (see Figure 2a) suggests that dog and cow are the outgroup to the primate clade. This is a common error in mammalian phylogeny reconstruction, and has been proven to be an effect of LBA [36]. Also, for the TPO group opossum is placed next to rat and mouse and not as the outgroup as expected, suggesting that the opossum and the rodents have similar rapid rates of evolution, see Figure 2a.

**Figure 3:**
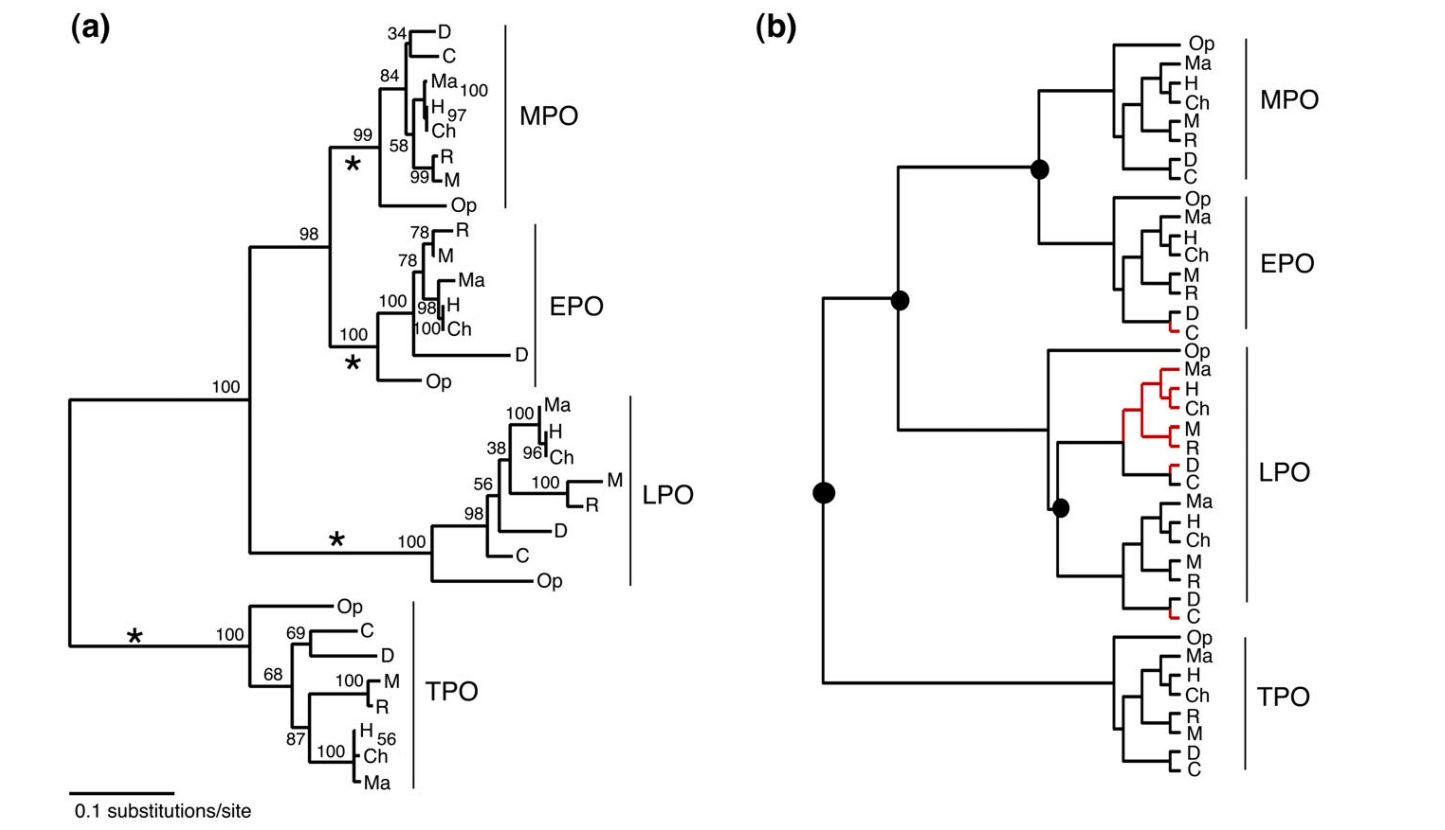
**Fully resolved mammalian heme peroxidase phylogeny with duplication and loss events depicted**. **(a) **Resolved ML tree for mammalian heme peroxidases. The bootstrap support values from 1000 replicates are shown on all nodes. The TPO primate clade appears here as a polytomy as the branch lengths are extremely short, however, this is in fact resolved with a low Bootstrap of 56%. The star symbol denotes those branches that were treated as foreground in the selection analysis. **(b) **The analysis of the resolved phylogeny using gene tree species tree reconciliation method implemented in GeneTree. The large filled circles represent gene duplication events, and the red branches indicate gene losses.

We adapted the site stripping method using the slow-evolving positions for each species in the MSA to reconstruct the phylogeny, while still retaining adequate amounts of signal [29]. This approach is similar to the '*Slow-Fast Method*' [37] and is therefore an approximate method that removes noise from the data by removing those sites that are most likely to contain homoplasy and focusing on the more evolutionary informative positions for phylogeny reconstruction. Each site within the MSA was classified according to rates of evolution (estimated using ML based on a fixed phylogenetic tree). To determine what number of categories to remove, we progressively stripped each category from the most rapidly evolving sites to the most slowly across the entire MSA. We also combined removal of the fastest and slowest sites from the dataset in our analysis, this was initially performed with the PXDN data included, see Figure 1b. Each time a category was removed the phylogenetic tree was estimated from the remaining MSA using ML. The ideal tree was created by pruning the mammalian supertree as published by Murphy *et al*. [33] (with the inclusion of chicken) and is depicted in Figure 1a. The difference between each site-stripped phylogeny and the ideal phylogeny was calculated using a nodal distance calculation RMSD [38], see Figure 1b. From Figure 1b, it is seen that the removal of rapidly evolving sites gradually removes the noise from the data and the remaining signal moves towards the canonical species phylogeny [33]. For the dataset consisting of MHPs and PXDN sequences, the RMSD value reaches a minimum at the removal of 4 site categories (8, 7, 6 and 5) leaving a MSA of length 850 sites (including gaps/missing data), see Figure 2b for resultant topology, after this point the RMSD values rise, see Figure 1b. It is important to note that the slowest evolving positions can be misleading particularly with excessive removal of sites, as the number of characters for reconstruction will decrease with every cycle, therefore caution must be taken in applying this method. This analysis was also performed on the dataset containing only MHP sequences, and the RMSD value reaches a minimum at the removal of 3 site categories (8,7, and 6) leaving a MSA of length 613 sites (including gaps/missing data), see Figure 3a for resultant topology. The reduced MSA for MHP data is given in Additional file 1 and the corresponding TOPD results are given in Additional file 2. The nodal distance (RMSD) calculation is based entirely on the branching pattern and hence does not account for evolutionary rate variation across the phylogeny. Using this site-stripped MSA the phylogeny was estimated using both MrBayes and MultiPhyl methods, both of which produced identical phylogenies*. (*We note here that the one exception, using the Bayesian reconstruction method, was the TPO primate monophyly was not fully resolved in the TPO clade but instead supported a human-chimp-macaque polytomy.)

All gene duplication events were verified using gene tree – species tree reconciliation. We analysed the resolved MHP phylogeny (Figure 3a), and identified in total 4 duplication events and 4 losses. This method over prescribes gene losses as in the case of EPO, where the sequence data was not available and therefore is assumed to be a loss. There is an LPO specific duplication event predicted, see Figure 3b. Our results show differential retention and loss in the LPO lineage following this gene duplication event resulting in the cow species retaining an alternative duplicate copy to the other mammals in the dataset, as shown in Figure 3b. This method must be used with caution as it does not take into account rate heterogeneity amongst species or sites in the data, and relies solely on the topology. However, reciprocal BLAST analysis of the cow sequence against the other mammal genomes identifies this sequence as an ortholog.

### Functional Diversity and Evolution of Specificity

We wished to test the hypothesis that following the gene duplication events in the MHPs (as resolved in this study), selective forces – specifically positive selection – have contributed to the observed changes in function in each of the 4 major groups of MHPs. Tests for heterogeneous selective pressures were carried out on the resolved phylogeny using the evolutionary models implemented in PAML 3.15 [39] and the complete MSA. The Dn/Ds ratios were estimated in a likelihood framework at both site-specific and lineage-specific levels. A total of seven tests of significance were carried out using χ^2 ^tests of significance, five site-specific comparisons and two branch-site comparisons were performed.

No positively selected sites were estimated for the one ratio model (see Additional file 3). Strong purifying selection across sites was indicated with an ω of 0.1516. However, this model is a poor fit for the data (ln*L *= -34417.1085). Positive selection was tested in a site-specific manner across the dataset using the site models; M1 (neutral), M2 (selection), M3 discrete (k = 2), M3 discrete (k = 3), M7 (beta), M8 (beta & omega > 1) and M8a (beta & omega = 1). The results of the site-specific analysis are shown in Additional file 3.

Poor likelihood values were achieved using the site-specific models of evolution, however, the most complex site-specific model used, M8 yielded significant results when it was tested with its null model M8a. A small proportion of sites are under relaxed positive selection (Additional file 3). Through the use of Bayesian estimations, four positively selected sites have been identified across the alignment, with posterior probability (PP) > 0.50.

Results of the branch-site model B with each of the families individually labeled as foreground are shown here in Table 2; see Figure 3a for corresponding foreground branches. (Results for model A are given in Additional files 4 and 5). To determine whether there is rate heterogeneity along different branches in the phylogeny, we compared models allowing for only site-specific evolution with those allowing for branch-site specific evolution (i.e. M3 K = 2 with Model B and M1 with Model A). Following LRT analysis it was found that both models A and B were significant following χ^2 ^test with two degrees of freedom. The likelihood score from model B for each family had improved significantly from those obtained using model A, as a result, model B was determined as the best fit model in each case tested and these results are summarized in Table 2. Positively selected sites identified with model B were estimated using the Naïve Empirical Bayes (NEB) method [40]. The results of which are discussed now in detail.

**Table 2:** Parameter estimates and likelihood scores for branch-site model, model B.

Model	P	L	Estimates of parameters	Positively selected sites
**MPO** Model B	5	-33655.0405	p_0 _= 0.4975, p_1 _= 0.4553, (p_2 _= 0.0246, p_3 _= 0.0225) *Background*: ω_0 _= 0.0458, ω_1 _= 0.3307, ω_2 _= 0.0458, ω_3 _= 0.3307 *Foreground*: ω_0 _= 0.0458, ω_1 _= 0.3307, ω_2 _= 251.6783, ω_3 _= 251.6783	**Foreground**: NEB 19 > 0.50 2 > 0.95 1 > 0.99
**EPO** Model B	5	-33647.5634	p_0 _= 0.4967 p_1 _= 0.4469, (p_2 _= 0.0297, p_3 _= 0.0267) *Background*: ω_0 _= 0.0464, ω_1 _= 0.3322, ω_2 _= 0.0464, ω_3 _= 0.3322 *Foreground*: ω_0 _= 0.0464, ω_1 _= 0.3322, ω_2 _= 774.6323, ω_3 _= 774.6323	**Foreground**: NEB 28 > 0.50 6 > 0.95 4 > 0.99
**LPO** Model B	5	-33627.3508	p_0 _= 0.4431, p_1 _= 0.3884, (p_2 _= 0.0898, p_3 _= 0.0787) *Background*: ω_0 _= 0.0470, ω_1 _= 0.3414, ω_2 _= 0.0470, ω_3 _= 0.3414 *Foreground*: ω_0 _= 0.0470, ω_1 _= 0.3414, ω_2 _= 82.8559, ω_3 _= 82.8559	**Foreground**: NEB 96 > 0.50 18 > 0.95 11 > 0.99
**TPO** Model B	5	-33639.5793	p_0 _= 0.4358, p_1 _= 0.3690, (p_2 _= 0.1057, p_3 _= 0.0895) *Background*: ω_0 _= 0.0479, ω_1 _= 0.3468, ω_2 _= 0.0479, ω_3 _= 0.3468 *Foreground*: ω_0 _= 0.0479, ω_1 _= 0.3468, ω_2 _= 999.0000, ω_3 _= 999.0000	**Foreground**: NEB 82 > 0.50 8 > 0.95

Model B allows each foreground lineage to be tested independently of all other lineages, hence the four clusters (MPO, EPO, LPO, TPO – each in turn treated as foreground), and estimates 5 parameters (P) in total. p_0_, p_1_, p_2 _and p_3 _are proportions of sites in the dataset with the corresponding ω value, i.e, ω_0_, ω_1_, ω_2 _and ω_3 _for the foreground and the background lineages independently. The final column gives the estimated number of sites with posterior probabilities of greater than 0.50 of belonging to the positively selected category. Note: NEB: Naïve Empirical Bayes analysis.

Our results show that following gene duplication, each individual type of MHP has undergone positive selection in amino acid residues that are unique to that type of MHP, see Table 2. As positive selection is closely associated with functional shift, we postulate that these positively selected sites have significantly contributed to the evolution of the functional diversity of these MHPs.

For the MPO superfamily, a total of 19 positively selected sites were identified (PP > 0.50). We have found functional information from the literature on 11 of these sites, these are now discussed: Position 80 (Arg) is located within the propeptide sequence and is under positive selection. Previous studies indicate that propeptide in MPO plays a key role in the processing and sorting of human MPO [41]. Position 568 is under positive selection and is next to the polymorphic site R569W, mutations in position 569 have been shown to suppress posttranslational processing in MPO [42]. The 2 positions with strongest support, PP > 0.95, are separated by 8 amino acid residues on the MPO heavy chain, they are Asn496 and Leu504. These 2 positions along with Tyr500 are in close proximity to the proximal heme ligand in MPO, His502 [3]. Position 259 (Leu) is located between two important distal residues, Gln257 and His261, involved in the formation of hydrogen bonds [3]. His261 has an important role in the formation of compound I, a redox intermediate of the peroxidase cycle [2]. A further four sites (Leu630, Gln633, Glu652; (primates Lys652) and Asn654 (primates Lys654) were identified as positively selected, PP > 0.70, these are located within a disulfide bond linking helices 19 and 22 on the MPO heavy chain. Disulfide bonds are associated with the folding and stability of proteins and as such are significant to the overall function of that protein [43].

For the EPO clade, 28 sites are positively selected, PP > 0.50. We have found functional information for 15 of these sites. One of these, Asp71, is located in the EPO propeptide. The inferred phylogeny, shown in Figure 3a, suggests that MPO and EPO are closely related enzymes, therefore it may be possible that the EPO propeptide may also be crucial for the function of EPO. The region separating the catalytic residues Arg377 and His474 [3], contains 8 positively selected sites (PP > 0.50). Arg377 is the conserved prominent distal amino acid associated with hydrogen bond formation. The proximal heme ligands His474 (EPO), His502 (MPO) and His468 (LPO), are conserved in all the MHPs [3,25]. Six of the 28 positively selected sites, Arg584, Gln588, Arg591, Ala618, Gly626 and Ala627, are located on the EPO heavy chain within a single disulfide bond region, this would suggest that they are structurally and functionally important to EPO. Position 441 has been identified as under positive selection, this residue has also been noted as being polymorphic in the human population (Lys/Thr).

There are 18 positively selected sites for the LPO group (PP > 0.95). We have found functional information on 13 of these sites. Residues Glu72, Asn87 and Trp91 are found in the LPO propeptide sequence and have a probability of greater than 0.95 of being positively selected. Residues Asn255, Phe282, Ser312, Ser352 and Glu355 are all located in the disulfide bond region (PP > 0.95). From biochemical analysis both Arg372 (Arg377 in EPO) and His468 are believed to have catalytic properties, and are conserved in the MHPs [3,25]. We find positive selection in His376 (PP > 0.99) just four amino acids downstream of the first of these catalytic residues (Arg372), interestingly this site is specific to the primate lineage. Also we have detected positive selection in Glu470 (PP > 0.98) adjacent to the second catalytic site (His468). We have also detected positive selection in Asp700 which is a known genetic variant and Glu240 and Gln245 that are located to the right and left of a known human polymorphism A244T.

With the TPO clade treated as foreground, 8 sites are positively selected, PP > 0.95. Of these 8 sites, 6 are missing in the alternatively spliced TPO isoform 5, which exhibits incorrect protein folding [44]. Asp228 (PP > 0.95), Ala232 and Ala242 (both PP > 0.50) are in the region of the TPO active site His239. Glu378 has also been identified as a novel mutational site (E378K) associated with the common inherited deficiency total iodide organification defect (TIOD) and is under positive selection in our analysis [45].

Independent analysis for positive selection using DIVERGE [46] software further supports our findings, see Table 3 for summary of results. Values greater than zero for the coefficient of functional divergence, *θ*, indicate a functional shift between clusters. Rate heterogeneity among sites varies with respect to the gamma distribution (α). We estimated *θ *for each of the four MHP clusters. This analysis shows significant functional constraints among the four MHP clades, with the null hypothesis *θ *= 0 being rejected for all clusters analysed. The analysis of closely related MPO and EPO clusters result in the lowest *θ *value (0.2833 +/- 0.0837), and both have microbicidal activity (Table 1). *θ *increases at least 1.5 fold for the more distantly related/functionally divergent clusters. These results provide statistical evidence of the diverse functions of these MHP enzymes.

**Table 3:** Summary of results of analysis using DIVERGE software.

	MPO/EPO	MPO/LPO	MPO/TPO	EPO/LPO	EPO/TPO	LPO/TPO
θ **ML**	0.2832	0.4504	0.4984	0.4552	0.4304	0.4280
**SE θ**	0.0837	0.0744	0.0783	0.1021	0.0950	0.0756
**LRT θ**	11.4512	36.6860	40.4815	19.8713	20.5223	32.0448
α **ML**	0.3034	0.4221	0.4172	0.4863	0.4654	0.5413

Each cluster analysed is shown in the columns of the table. **θ ML**: Coefficient of functional divergence. **SE θ**: Standard error of the estimate Theta. **LRT θ**: 2 log-likelihood-ratio against the null hypothesis of **θ **= 0. **α ML**: Gamma shape parameter for rate variation among sites.

**Figure 4:**
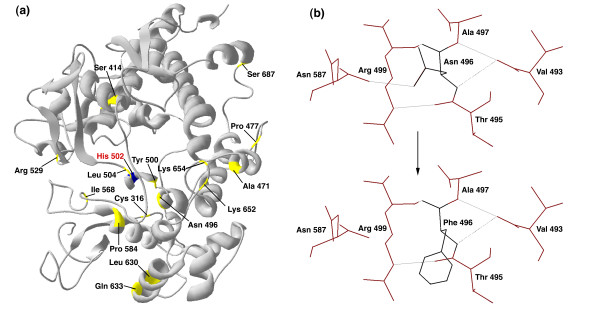
**Location of positively selected sites in the MPO structure and their effect on bonding within the structure**. **(a) **3-D structure of the human MPO sequence, highlighted in gold are those sites that are positively selected in MPO, in blue is the heme binding site. **(b) **Example of the affect on hydrogen bonding of one such mutation at positively selected position 496 in human MPO from Asparagine to Phenylalanine.

We further test the relationship between positive/directional selection and functional shift by analyzing the effect of these substitutions on the MPO 3D structure, see Figure 4. Modeling the MPO human sequence using SwissModel and using the mutate tool in DeepView v3.7, we have performed *in silico *site directed mutagenesis on those sites identified in our study as being positively selected [47,48]. The structure with positively selected sites and the heme binding site is shown in Figure 4a. We find that mutating these positions from their positively selected state to the ancestral state causes a variety of effects on the hydrogen bond formation within the 3D structure, see Table 4 for a summary of the effects on hydrogen bonds. Hydrogen bonds play an important role in maintaining the structural integrity of a protein, any disruption of such forces is likely to upset the balance between the structural and functional dynamics [49]. On mutating each of these 19 positively selected amino acids we find that 4 bonds are lost and 4 are independently gained in the protein, for summary see Table 4. For the mutations: N496F, Y500F, and L504T, the positions of the losses and gains of hydrogen bonds are significant as these amino acid are in close proximity to the proximal heme ligand His502, shown in Figure 4a. The mutation from leucine to threonine at position 504 results in the formation of an additional hydrogen bond between Gly501 and Leu504. Gly501 is directly bound to the proximal heme ligand. In addition, the N496F mutation illustrated in Figure 4b, results in the loss of the hydrogen bond with Asn587. The Asn587 and His502 are connected by a hydrogen bond [3]. The loss of the hydrogen bond, as a result of the mutation at position 496, is likely to affect the structural integrity of the link between Asn587 and His502. Disruption to the hydrogen bonds in this catalytically important region may have direct implications for functional divergence of the MPO enzyme. The A471R mutation results in an increase in the number of hydrogen bonds associated with this position. This position is upstream from Asn483 which is thought to be responsible for MPOs dimer interaction [3]. The mutation from cysteine to serine at position 316 results in the formation of a hydrogen bond with Gln329 and the loss of one of the bonds to Asp593, see Table 4. Cys316 is next to the single disulphide bridge (Cys319) that connects MPOs symmetry-related halves [3]. The C316S mutation may potentially disrupt this disulphide bridge.

**Table 4:** Summary of results from SwissModel analysis of positively selected sites.

Mutation	Posterior Probability	Affect on Hydrogen Bond
**C316S**	0.815	-/+
**S414A**	0.600	-
**A471R**	0.738	+
**P477G**	0.948	=
**N496F**	0.999	-
**Y500F**	0.731	-
**L504T**	0.970	+
**R529E**	0.657	+
**I568L**	0.686	=
**P584A**	0.949	=
**L630F**	0.767	=
**Q633L**	0.737	=
**L652V**	0.840	=
**L654G**	0.921	=
**S687T**	0.648	+

Mutation from positively selected site in MPO (using human model) to the amino acid present in EPO, LPO and TPO at that position (in cases where there was conflict the majority rule consensus at that position was taken). Posterior Probability values extracted using NEB analysis in model B Codeml. Effect on H-Bonds is classified as "+" if an increase in the number of bonds with positively selected amino acid, "-" if a hydrogen bond or a number of hydrogen bonds were lost with the positively selected site, and "=" refers to no affect on the hydrogen bond with the positively selected site.

## Discussion and Conclusion

The MHPs are a functionally diverse family of enzymes which are implicated in a variety of inflammatory and neurodegenerative diseases such as asthma and AD respectively. In this study the evolutionary history of the four major groups of MHPs; MPO, EPO, LPO and TPO, was investigated allowing for the analysis of their functional diversity.

Initial ML and Bayesian phylogenies estimated here for the MHPs support previous biochemical studies [3,8,9]. From Figure 3 the order of gene duplication events can be traced, with an MPO-EPO-LPO MRCA arising from a gene duplication with extant TPO; then a further duplication event that gave rise to, (i) the MPO-EPO MRCA, and (ii), the lineage leading to extant LPO; and the final and most recent duplication of the MPO-EPO MRCA into extant MPO and EPO clades. PXDN is the outgroup to the MHP sequences and was included in the analysis to illustrate that TPO is the most ancestral MHP (Figure 2a). However, the species relationships estimated within these clearly defined clades were in disagreement with the previously resolved mammalian phylogeny [33].

Including all sites of the alignment in the analysis, we have shown that the major types of MHP form monophyletic clades and are therefore the result of gene duplication events prior to speciation of modern day mammals, see Figure 2(a). However, also evident from Figure 2(a), species with more similar generation times are clustered together, with species of shorter generation times and therefore more rapid rates of mutation assuming a basal position in the phylogeny. This observed branching pattern could be a result of LBA, incorrect ortholog prediction or hidden paralogy.

If a phylogeny is seen to approach the ideal by removing the most rapidly evolving sites, then we propose that LBA is most likely to have contributed to the misleading phylogeny. To test for the presence of LBA we calculated 8 categories of rates of evolution for all sites, from the most rapidly evolving to the most slowly evolving. We observed that the sequential removal of rapidly evolving categories of sites from the alignment decreased the difference, in terms of nodal distance RMSD, between the phylogeny produced and the ideal phylogeny. This occurred only for removal of the 4 fastest evolving categories of site from the alignment. Further removal after this point resulted in increased RMSD values between the phylogeny produced and the ideal. The MHP phylogeny shown in Figure 3(a), with maximum number of sites and minimum amount of noise. We propose that a possible reason for the presence of LBA in this dataset is the presence of taxa with vastly different generation times. The rodentia have previously been shown as "fast evolving" due to their short germ-line generation time, whereas species such as dogs and humans have longer germ-line generation times [27,28,50]. In any given dataset there are sites that are variable and sites that are invariable, this pattern is conserved across homologous sequences. In a dataset with a mixture of germ line generation times, the mutation rate in the species with shorter germ line generation times will be higher, because the number of cell divisions per unit time is greater. Therefore the number of mutations in the variable regions will increase for these species. The result is an LBA effect derived from having a mixture of long and short germ line generation times in the dataset, where the species with a short germ line generation time assumes a basal position in the phylogeny [26-28]. A number of approaches have been explored to systematically deal with fast evolving taxa the most popular include, (1) reconstructing the phylogeny based on slow evolving sites (applied here), (2) increasing the sample size, this is based on the assumption that increasing the sample size actually increases the number of slowly evolving positions, (3) decreasing the distance to the outgroup, and (4) using more accurate models of sequence change such as covarion derivatives.

Our gene tree – species tree reconciliation analysis has verified the duplication pattern amongst the MHPs. However, we believe that current methods of reconciliation such as the one used here may be biased towards inferring excess gene duplication and differential loss events, as is the case here. The method only considers the topology and not the corresponding alignment or any rate heterogeneity that may exist [51]. We would also like to highlight that the variation of the "*Slow-Fast" *method employed here is an approximate method for a complex evolutionary dynamic and is not without its limitations.

Using this fully resolved phylogeny, positively selected sites have been identified, through the use of Bayesian estimation, unique to all four MHPs; MPO, EPO, LPO and TPO. The majority of these sites are in close proximity to catalytically important residues, suggesting that they may potentially be linked to functional shifts across the MHPs. The conserved proximal histidines in close proximity to sites under positive selection in MPO, EPO and LPO are crucial in preserving the redox properties of the heme iron for catalysis [3]. The conserved distal histidines, also shown here to be in the vicinity of positively selected sites, act as both proton acceptors and donor to oxygen during the formation of Compound 1, which is an integral step in the peroxidase pathway [3]. A number sites identified under positive selection are located in disulphide bond regions, which are believed to be crucial to the structure and function of a protein. Disruption of such regions can be detrimental to the enzymatic stability and activity [43,52]. In particular, six sites pertaining to the LPO family are linked to the same disulphide bond. This strongly suggests that these sites are associated with the unique function of LPO as they are not present in the two closely related families MPO and EPO. In the TPO analysis the majority of the sites with highest probability of being positively selected are located in exon 8 of the protein. Deletion of exon 8 results in misfolding of the TPO protein [44]. Exon 8 is also believed to be part of TPOs catalytic centre (exons 8, 9 and 10) [53]. TPO functional defects are strongly associated with TIOD and several deleterious mutations within this catalytic region have been reported [44,53-55]. We also find that one of our positively selected sites in TPO is associated directly with an inherited deficiency disorder [55].

Our detailed *in silico *site directed mutagenesis of the positively selected sites in MPO has shown that mutating these positions from their positively selected amino acid state to an alternative ancestral state results in loss/gain of hydrogen bonds between alternative amino acid positions for other sites in particular in the heme binding region of the MPO structure. The sites we have identified as positively selected in the MHPs have played a major role in the functioning of these enzymes as evidenced by mutational studies, proximity to active sites and catalytic residues, and inherited disorders.

The results of this study show for the first time from molecular sequence data (i) how this medically important group of enzymes are related to each other, and (ii) suggest that following gene duplication, positive selection has led to the functional diversity observed for the MHPs.

## Methods

### Sequence Data

Protein coding sequences for MHPs were retrieved from the Ensembl database for all available completed mammalian genomes using the pre-defined orthologues identified in Ensembl [56]. The mammalian genomes and the corresponding genome versions used for each of the major families in our dataset were as follows: *Homo sapiens *v42.36d; *Pan troglodytes *v42.21a; *Macaca mulatta *v42.10b; *Mus musculus *v42.36c; *Rattus norvegicus *v42.34l; *Canis familiaris *v42.2; *Bos taurus *v42.2e (no EPO sequence available), and, *Monodelphis domestica *v42.36c. Ensembl identifies orthologues by performing a genome-wide reciprocal WUBlastp+SmithWaterman search of each gene across all completed genomes. Multiple sequence alignment (MSA) is then performed using the MUSCLE software [57] and the best reciprocal hits following the sequence similarity search. The longest alternative transcript in each case was used. These sequences were combined into a single MHP dataset of 31 sequences. Two amino acid sequences representing the peroxidasin (PXDN) family, from the *Pan troglodytes *and the *Gallus gallus *genomes, were retrieved from the PeroxiBase database [31]. The sequence data are given in Table 5.

**Table 5:** Representative mammalian heme peroxidase sequences used in this study.

Superfamily	Species	Entry ID (Name)*/Gene ID	Length (aa)
MPO	*Homo sapiens*	ENSG00000005381	778
	*Pan troglodytes*	ENSPTRG00000009449	778
	*Macaca mulatta*	ENSMMUG00000002266	777
	*Mus musculus*	ENSMUSG00000009350	719
	*Rattus norvegicus*	ENSRNOG00000008310	719
	*Canis familiaris*	ENSCAFG00000017474	743
	*Bos taurus*	ENSBTAG00000012783	596
	*Monodelphis domestica*	ENSMODG00000014737	403

EPO	*Homo sapiens*	ENSG00000121053	716
	*Pan troglodytes*	ENSPTRG00000009446	716
	*Macaca mulatta*	ENSMMUG00000011973	717
	*Mus musculus*	ENSMUSG00000052234	717
	*Rattus norvegicus*	ENSRNOG00000008707	716
	*Canis familiaris*	ENSCAFG00000017456	752
	*Monodelphis domestica*	ENSMODG00000014755	725

LPO	*Homo sapiens*	ENSG00000167419	713
	*Pan troglodytes*	ENSPTRG00000009448	712
	*Macaca mulatta*	ENSMMUG00000002264	716
	*Mus musculus*	ENSMUSG00000009356	711
	*Rattus norvegicus*	ENSRNOG00000008422	710
	*Canis familiaris*	ENSCAFG00000024533	719
	*Bos taurus*	ENSBTAG00000012780	713
	*Monodelphis domestica*	ENSMODG00000014744	719

TPO	*Homo sapiens*	ENSG00000115705	934
	*Pan troglodytes*	ENSPTRG00000011610	857
	*Macaca mulatta*	ENSMMUG00000009662	839
	*Mus musculus*	ENSMUSG00000020673	915
	*Rattus norvegicus*	ENSRNOG00000004646	915
	*Canis familiaris*	ENSCAFG00000003217	932
	*Bos taurus*	ENSBTAG00000002567	869
	*Monodelphis domestica*	ENSMODG00000014296	872

PXDN	*Pan troglodytes*	5828 (PtroPxd01)*	1463
	*Gallus gallus*	4049 (GgaPxd01)*	1447

Note: * – Assigned entry ID and Name in the PeroxiBase database

The common names for the genomes used are; *Homo sapiens*: human, *Pan troglodytes*: chimp, *Macaca mulatta*: macaque, *Mus musculus*: mouse, *Rattus norvegicus*: rat, *Canis familiaris*: dog, *Bos taurus*: cow, *Monodelphis domestica*: opossum, *Gallus gallus*: chicken. aa: amino acid.

### Multiple Sequence Alignment

Each protein coding sequence in the MHP dataset was translated to amino acid using in-house translation software. This protein sequence dataset and the two PXDN sequences were combined to give a dataset of 33 sequences (complete dataset). Both MHP and "complete" datasets were aligned in ClustalW 1.8 [58] independently using default parameter settings. The corresponding nucleotide sequences for the MHP dataset were aligned with respect to the amino acid MSA with the use of in-house software to insert gaps in the protein coding sequence according to their positions in the amino acid alignment. The nucleotide and subsequent protein MSAs were manually edited by removing ambiguous regions from the alignment using the sequence alignment editor, Se-Al 2.0a11 [59]. The PXDN sequences served as an outgroup for the MHPs and therefore aided in determining the earliest diverging MHP.

### Site Stripping and Phylogeny Reconstruction

The phylogenetic tree for the dataset was estimated using Bayesian statistics implemented in MrBayes 3.1.2 [35]. The model of amino acid substitution used was JTT [60] because following model testing using MultiPhyl [34] this was the model that was best-fit to the data. Using 4 Markov chains for 400,000 generations, trees were sampled every 10 generations with the first 20,000 sampled trees discarded as burnin. The remaining trees samples were summarized on a majority rule consensus tree with clade supports given as Posterior Probabilities (PPs). ML trees were also inferred using the high-throughput phylogenomics webserver, MultiPhyl [34]. The ML tree was generated using the nearest neighbour interchange (NNI) tree search algorithm and 100 bootstrap replicates implemented in MultiPhyl [34] under the Akaike Information Criterion (AIC) statistic, the selected substitution model was JTT with invariable sites and a discrete gamma model of rate heterogeneity. This was repeated a total of 10 times to generate 1000 bootstrap replicates. (The Bayesian tree reconstruction methods were applied to the MHP dataset only).

The resulting phylogenies from both analyses (MrBayes and MultiPhyl) were then analysed for signatures of LBA. The rate of evolution at each site in the alignment was placed into one of 8 categories, 8 being the most rapidly evolving and 1 being the most conserved, using the maximum likelihood approach implemented in TreePuzzle 5.1 [61]. Sites were progressively removed from the protein MSA according to their evolutionary rate and the resultant trees were analysed for changes in topology.

Nine separate site-stripped alignments were constructed by successive removal of the most rapidly evolving sites [29]. The aforementioned Bayesian method was used to infer phylogenetic relationships for each of the nine alignments generated. The ML phylogeny was also estimated for each of the site-stripped alignments from the model of best-fit following hierarchical likelihood ratio tests (hLRTs) of alternative models implemented in MultiPhyl [34].

### Nodal Distance Analysis

The pruned nodal distance method implemented in TOPD/FMTS v3.3 [38] was used to calculate the distance between each of the site-stripped trees and the ideal tree. The ideal tree was generated by pruning the resolved mammalian phylogeny [33] to represent those taxa present. A distance matrix is calculated for both the site-stripped phylogeny and the ideal phylogeny by counting the number of nodes that separate every taxon from every other taxon on the tree. Using the root means squared deviation (RMSD) implemented in the TOPD/FMTS v3.3 [38] software package, the RMSD between the site-stripped phylogeny matrix and the ideal phylogeny matrix is calculated. A RMSD value of zero indicates that the two trees being compared are identical.

### Gene Tree – Species Tree Reconciliation

Following nodal distance analysis, the gene phylogeny with the lowest RMSD value (for the MHP sequences alone), and the species tree were examined for gene duplication and loss events using the default settings for gene tree – species tree reconciliation implemented in GeneTree 1.3.0 [62].

### Selective Pressure Analysis

Analysis of variation in selective pressure following gene duplication in the MHPs was carried out using codon substitution models implemented in PAML 3.15 [39]. Both site-specific and branch-site specific models were applied. The models used for this analysis allow for heterogeneous nonsynonymous-to-synonymous rate ratios (ω = *Dn/Ds*) across sites and amongst branches/lineages.

An ω-value > 1 indicates positive selection, ω < 1, purifying selection and neutral evolution when ω = 1. The statistically significant model for the data was selected using a series of LRTs to compare models and their more parameter rich extensions. Tests of significance were carried out using χ^2 ^tests of significance, the comparisons performed were; M0 (one ratio) with M3(*k *= 2)(discrete), M1(neutral) with M2(selection), M3(*k *= 2) with M3(*k *= 3) discrete models, M7 (beta) with M8 (beta & omega > 1), M8 (beta & omega > 1) with the null hypothesis M8a (beta & omega = 1), M1 with model A (branch-site) and finally M3(*k *= 2) with model B (branch-site). The models and approach taken here have been described previously [39,63].

The probability (PP) of a specific amino acid site belonging to the positively selected category is estimated using the empirical Bayes method for each superfamily individually [40,64,65].

### Functional Divergence analysis

Using the MHP gene phylogeny with the lowest RMSD value, each of the four MHPs were selected as independent clusters. Using the MHP protein MSA and this MHP gene phylogeny, statistical analysis implemented in the software DIVERGE v 1.04 [66,46], was used to estimate the coefficient of functional divergence (theta ML or *θ*) for all pairs of clusters. The following are the clusters used in the analysis are taken from the resolved phylogeny (from Figure 3a) (1) MPO Cluster, (2) EPO Cluster, (3) LPO Cluster, and (4) TPO Cluster.

### 3D Modeling and In Silico Mutational Analysis

Homology modeling was performed using the human representative sequence for the MPO family and the first approach mode implemented by the homology-modeling server, SWISS-MODEL [48]. The structure was modeled using the crystal structure of bromide-bound human MPO isoform C (PDB accession code 1d2vC). The positively selected sites identified from the PAML 3.15 (Yang 1997) analysis were highlighted (in gold) on the 3D structure generated using DeepView v3.7 [47]. The conserved proximal heme ligand (His 502) was also highlighted (in blue) on the 3D model. *In silico *mutational analysis on these positively sites was carried out and their subsequent affect on hydrogen bonding was assessed using DeepView v3.7 [47].

## Abbreviations

AD: Alzheimer's disease, AIC: Akaike Information Criterion, BEB: Bayes Empirical Bayes, DDC: Duplication-Degeneration-Complementation, Dn: nonsynonymous substitutions per nonsynonymous site, Ds: synonymous substitutions per synonymous site, EPO: Eosinophil peroxidase, hLRT: hierarchical Likelihood Ratio Test, JTT: Jones, Taylor and Thornton, LBA: Long Branch Attraction, LPO: Lactoperoxidase, LRT: Likelihood Ratio Test, MHP: Mammalian Heme Peroxidase, ML: Maximum Likelihood, MPO: Myeloperoxidase, MRCA: Most Recent Common Ancestor, MSA: Multiple Sequence Alignment, NEB: Naïve Empirical Bayes, NNI: Nearest Neighbour Interchange, PDB: Protein Data Bank, PP: Posterior Probability, PXDN: Peroxidasin, RMSD: Root Mean Squared Deviation, TIOD: Total Iodide Organification Defect, TPO: Thyroid peroxidase.

## Authors' contributions

NBL carried out the alignment construction, phylogenetic and selection analysis and participated in drafting the manuscript. MJO'C designed and co-ordinated the study, and was involved in phylogeny reconstruction, selection analysis and statistical analysis, data quality control and conceived of the study. BO'C and CÓ'F were involved in the co-ordination of the study, participated in data management, contributed to the biochemical interpretation of the data and helped to draft the manuscript. All authors read and approved the final manuscript.

## Supplementary Material

Additional file 1 **The resultant site stripped multiple sequence alignment of MHP sequences**. This figure depicts the multiple sequence alignment that was selected following RMSD analysis. This alignment has sites of rate category 8, 7, and 6 removed. Click here for file

Additional file 2 **RMSD nodal distance between each site-stripped MHP phylogeny and the ideal phylogeny**. This table summarizes the results of the statistical comparison (RMSD) of the ideal phylogeny with each site stripped phylogeny. Values closer to zero are closer to complete agreement, the alignment with site categories 8 through to 6 removed, is the phylogeny closest to ideal. Click here for file

Additional file 3 **Parameter estimates and likelihood scores of one ratio and site-specific models**. The data presented in this table are the results of ML analysis of site specific evolutionary models applied to the MHP alignment. The name of the model is given in column 1, the number of parameters estimated is given in column 2, the Log likelihood value in column 3, and the parameter estimates in column 4 and 5. Click here for file

Additional file 4 **Parameter estimates and likelihood scores for branch-site models: MPO and EPO clades**. This table summarizes the results of ML analysis on the MHP data, using branch specific models of evolution. The MPO and EPO clades are treated as foreground lineages independently and all other peroxidase clades as background. The LRTs are performed between model A and M1 and model B and M3K2 from Additional file 3. Click here for file

Additional file 5 **Parameter estimates and likelihood scores for branch-site models: LPO and TPO clades**. This table summarizes the results of ML analysis on the MHP data, using branch specific models of evolution. The LPO and TPO clades are treated as foreground lineages independently and all other peroxidase clades as background. The LRTs are performed between model A and M1 and model B and M3K2 from Additional file 3. Click here for file
