# Supplementary material for: The phylogeny of the mammalian heme peroxidases and the evolution of their diverse functions
Source: BMC Evol Biol. 2008 Mar 27;8:101. doi: 10.1186/1471-2148-8-101 (PMC2315650; doi:10.1186/1471-2148-8-101)
Supplement: Additional file 1 — The resultant site stripped multiple sequence alignment of MHP sequences. This figure depicts the multiple sequence alignment that was selected following RMSD analysis. This alignment has sites of rate category 8, 7, and 6 removed. [file 1471-2148-8-101-S1.pdf]

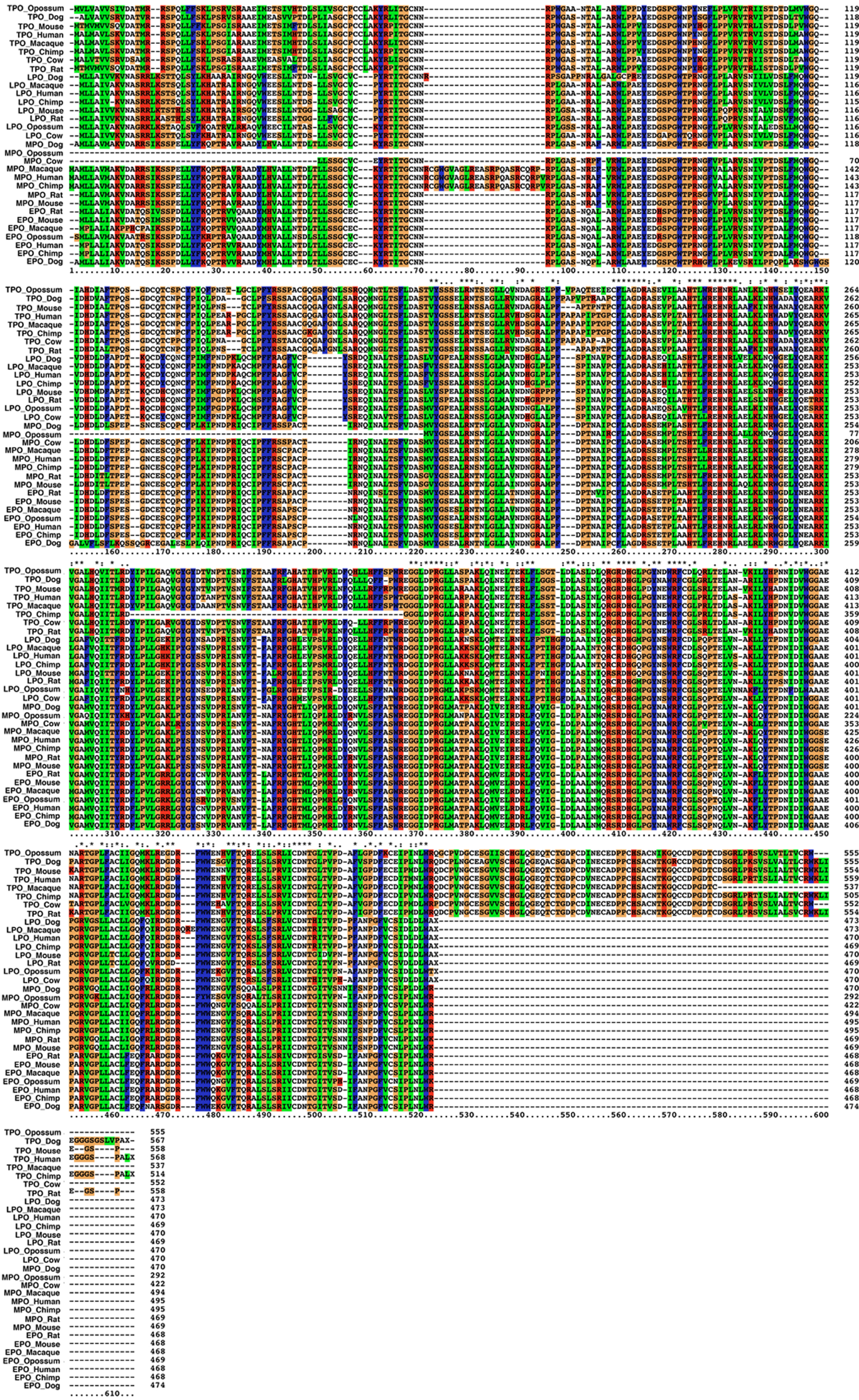

TPO_Opossum
TPO_Dog
TPO_Mouse
TPO_Human
TPO_Macaque
TPO_Chimp
TPO_Cow
TPO_Rat
LPO_Dog
LPO_Macaque
LPO_Human
LPO_Chimp
LPO_Mouse
LPO_Rat
LPO_Opossum
LPO_Cow
MPO_Dog
MPO_Opossum
MPO_Cow
MPO_Macaque
MPO_Human
MPO_Chimp
MPO_Rat
MPO_Mouse
EPO_Rat
EPO_Mouse
EPO_Macaque
EPO_Opossum
EPO_Human
EPO_Chimp
EPO_Dog
