# Supplementary material for: The phylogeny of the mammalian heme peroxidases and the evolution of their diverse functions
Source: BMC Evol Biol. 2008 Mar 27;8:101. doi: 10.1186/1471-2148-8-101 (PMC2315650; doi:10.1186/1471-2148-8-101)
Supplement: Additional file 2 — RMSD nodal distance between each site-stripped MHP phylogeny and the ideal phylogeny. This table summarizes the results of the statistical comparison (RMSD) of the ideal phylogeny with each site stripped phylogeny. Values closer to zero are closer to complete agreement, the alignment with site categories 8 through to 6 removed, is the phylogeny closest to ideal. [file 1471-2148-8-101-S2.doc]

**Additional File 2 :** **RMSD nodal distance between each site-stripped MHP phylogeny and the ideal phylogeny.**

|  | **Site Categories Removed** | | | | | | | | | |
| --- | --- | --- | --- | --- | --- | --- | --- | --- | --- | --- |
|  | **None** | **8 + 1** | **1** | **8** | **8 - 7** | **8 - 6** | **8 - 5** | **8 - 4** | **8 - 3** | **8 - 2** |
| **RMSD** | 1.2852 | 1.2217 | 1.2217 | 1.2217 | 0.8701 | 0.5680 | 1.1110 | 1.3034 | 1.5098 | 1.6422 |

None refers to the complete MHP MSA; 8 + 1: site categories 8 and 1 (i.e. the fastest and slowest) removed; 1: site category 1 removed from the MHP MSA; 8: site category 8 removed from the MHP MSA; 8, 7: categories 8 and 7 removed from the MHP MSA and so on up to the final column that contains only the most slowly evolving category of site.
