# Supplementary material for: The phylogeny of the mammalian heme peroxidases and the evolution of their diverse functions
Source: BMC Evol Biol. 2008 Mar 27;8:101. doi: 10.1186/1471-2148-8-101 (PMC2315650; doi:10.1186/1471-2148-8-101)
Supplement: Additional file 3 — Parameter estimates and likelihood scores of one ratio and site-specific models. The data presented in this table are the results of ML analysis of site specific evolutionary models applied to the MHP alignment. The name of the model is given in column 1, the number of parameters estimated is given in column 2, the Log likelihood value in column 3, and the parameter estimates in column 4 and 5. [file 1471-2148-8-101-S3.doc]

Additional File 3: Parameter estimates and likelihood scores of one ratio and site-specific models.

| **Model** | **P** | **L** | **Estimates of parameters** | **Positively**  **selected sites** |
| --- | --- | --- | --- | --- |
| M0 : one ratio | 1 | -34417.1085 | ω = 0.1516 | None |
| **Site-specific:**  M1:Neutral | 1 | -33999.1059 | p_0_= 0.7685 | Not allowed |
| M2:Selection | 4 | -33999.0008 | p_0_= 0.7685, p_1_= 0.2306  (p_2_= 0.0009), ω_2_= 1.0000 | None |
| M3:Discrete(K = 2) | 3 | -33666.6464 | p_0_= 0.5205, (p_1_= 0.4795)  ω_0_= 0.0464, ω_1_= 0.3311 | None |
| M3:Discrete(K = 3) | 5 | -33555.0100 | p_0_= 0.2272, p_1_= 0.4418, p_2_= 0.3310  ω_0_= 0.0062, ω_1_= 0.1124, ω_2_= 0.4242 | None |
| M7: Beta | 2 | -33545.1089 | p= 0.5950, q= 2.3340 | Not allowed |
| M8: Beta&Omega > 1 | 4 | -33540.9163 | p_0_= 0.9849, p = 0.6204, q = 2.6031  (p_1_= 0.0152 ), ω = 2.0778 | BEB  4 > 0.50 |
| M8a: Beta&Omega = 1 | 3 | -33542.7233 | p_0_= 0.9733, p = 0.6366, q = 2.801  (p_1_= 0.0268 ), ω = 1.0000 | Not allowed |

*Note: BEB: Bayes Empirical Bayes analysis*
