# Supplementary material for: The phylogeny of the mammalian heme peroxidases and the evolution of their diverse functions
Source: BMC Evol Biol. 2008 Mar 27;8:101. doi: 10.1186/1471-2148-8-101 (PMC2315650; doi:10.1186/1471-2148-8-101)
Supplement: Additional file 5 — Parameter estimates and likelihood scores for branch-site models: LPO and TPO clades. This table summarizes the results of ML analysis on the MHP data, using branch specific models of evolution. The LPO and TPO clades are treated as foreground lineages independently and all other peroxidase clades as background. The LRTs are performed between model A and M1 and model B and M3K2 from Additional file 3. [file 1471-2148-8-101-S5.doc]

**Additional File 5: Parameter estimates and likelihood scores for branch-site models: LPO and TPO clades.**

| **Model** | **P** | **L** | **Estimates of parameters** | **Positively**  **selected sites** |
| --- | --- | --- | --- | --- |
| **Branch-site**  Model A | 4 | **LPO**  -33923.5023 | p_0_= 0.6117, p_1_= 0.1814, (p_2_= 0.1596, p_3_= 0.0473)  *Background:*  ω_0_= 0.1119, ω_1_= 1.0000, ω_2_= 0.1119, ω_3_= 1.0000  *Foreground:*  ω_0_= 0.1119, ω_1_= 1.0000, ω_2_= 500.2431, ω_3_= 500.2431 | **Foreground:**  BEB  537 > 0.50  42 > 0.95  12 > 0.99 |
| Model B | 5 | -33627.3508 | p_0_= 0.4431, p_1_= 0.3884, (p_2_= 0.0898, p_3_= 0.0787)  *Background:*  ω_0_= 0.0470, ω_1_= 0.3414, ω_2_= 0.0470, ω_3_= 0.3414  *Foreground:*  ω_0_= 0.0470, ω_1_= 0.3414, ω_2_= 82.8559, ω_3_= 82.8559 | **Foreground:**  NEB  96 > 0.50  18 > 0.95  11 > 0.99 |
| **Branch-site**  Model A | 4 | **TPO**  -33928.0276 | p_0_= 0.5278, p_1_= 0.1497, (p_2_= 0.2513, p_3_= 0.0713)  *Background:*  ω_0_= 0.1134, ω_1_= 1.0000, ω_2_= 0.1134, ω_3_= 1.0000  *Foreground:*  ω_0_= 0.1134, ω_1_= 1.0000, ω_2_= 160.6529, ω_3_= 160.6529 | **Foreground:**  BEB  218 > 0.50  43 > 0.95  3 > 0.99 |
| Model B | 5 | -33639.5793 | p_0_= 0.4358, p_1_= 0.3690, (p_2_= 0.1057, p_3_= 0.0895)  *Background:*  ω_0_= 0.0479, ω_1_= 0.3468, ω_2_= 0.0479, ω_3_= 0.3468  *Foreground:*  ω_0_=0.0479, ω_1_= 0.3468, ω_2_= 999.0000, ω_3_= 999.0000 | **Foreground:**  NEB  82 > 0.50  8 > 0.95 |

*Note: NEB: Naive Empirical Bayes analysis, BEB: Bayes Empirical Bayes analysis (BEB results used where available)*
